# Blood adenosine triphosphate luminometry and dynamic viscoelastic coagulometry reference values in healthy newborn dairy calves

**DOI:** 10.3168/jdsc.2025-0977

**Published:** 2026-02-06

**Authors:** Laura Van Driessche, Gilles Fecteau, Claire Vergneau-Grosset, Sébastien Buczinski

**Affiliations:** Department of Clinical Sciences, Faculty of Veterinary Medicine, Université de Montréal, Saint-Hyacinthe, J2S 2M2

## Abstract

The primary objective of this study was to determine reference blood values for ATP luminometry and dynamic viscoelastic coagulometry analysis in healthy newborn dairy calves in order to evaluate their potential usefulness for future on-farm detection of sepsis. Secondary methodological objectives were to assess whether ATP luminometry results were influenced by blood collection method, blood matrix (serum, plasma or whole blood), or the addition of a stabilizing solution aimed at minimizing baseline relative light unit (RLU) values. Groups of 21 and 19 healthy calves were included in the study for ATP luminometry and dynamic viscoelastic coagulometry analysis, respectively. We performed ATP measurements using Hygiena AquaSnap Total and AquaSnap Free swabs across the different blood matrices, with and without stabilizing solution. Hemoglobin concentration and hematocrit were measured to account for potential effects of hemolysis and dilution on ATP results. No significant differences in RLU values were observed between sampling methods. Baseline ATP luminometry values were high across all matrices, with median RLU values of 5,062 for serum, 7,198 for plasma, and 7,547 for whole blood, and substantial interindividual variability, particularly for serum and plasma samples. The addition of stabilizing solution did not reduce baseline RLU values, and neither hemoglobin concentration nor hematocrit allowed for meaningful normalization between individuals. In contrast, dynamic viscoelastic coagulometry demonstrated narrow reference ranges and low interindividual variability, with median (interquartile range) values of 172 (154–192) seconds for activated clotting time, 25 (18–34) units/min for clot rate, and 3.30 (2.70–3.90) units for platelet function. Overall, these findings indicated that ATP luminometry, under the conditions evaluated, shows practical and methodological constraints. Conversely, dynamic viscoelastic coagulometry analysis shows promising analytical stability and warrants further investigation in clinically septic calves to determine its potential for early on-farm sepsis detection in newborn dairy calves.

## Highlights

• Sonoclot analysis delivered consistent blood values with low interindividual variation.
• Conventional or septic collection of blood does not influence RLU measurements.
• Serum, plasma, and whole blood give high RLU values or a high variation.
• Stabilizing solution does not lower RLU values in plasma or whole blood.
• Sonoclot, but not luminometry, shows potential as an indicator for on-farm sepsis in calves.

## Graphical Abstract

**Figure:**
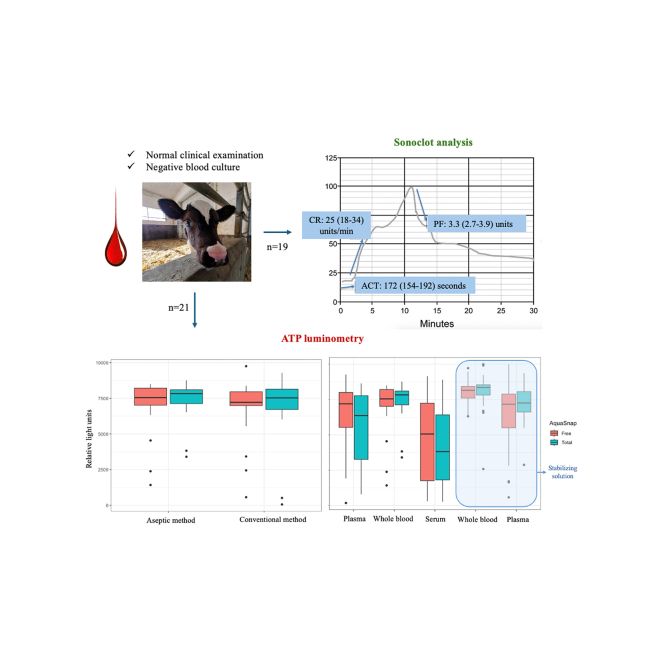
**Summary:** Reference blood values for adenosine triphosphate (ATP) luminometry and dynamic viscoelastic coagulometry (Sonoclot, Sienco) analysis in healthy newborn dairy calves were determined to assess the future potential of both techniques as a fast and reliable indicator for sepsis on the farm. Different sampling methods and blood matrices were investigated for ATP luminometry to achieve the lowest relative light unit (RLU) values possible to facilitate future research. For ATP luminometry, no difference in RLU values was found between the aseptic and conventional method of sampling. Differences in RLU values were found between different blood matrices (plasma, serum, and whole blood), but overall values were high, with a large variation. Adding stabilizing solution did not contribute to lowering RLU values or reducing variation between individuals. Sonoclot analysis showed consistent results with a small variation between individuals. These results suggest that Sonoclot analysis seems to have potential to be used as an on-farm technique for detecting possible sepsis in calves. ACT = activated clotting time; CR = clot rate; PF = platelet function.

Sepsis, an acute inflammatory response of the organism caused by a microbial infection in the blood, is one of the leading causes of mortality in young calves (Pardon and Deprez, 2018). An appropriate and prompt antimicrobial treatment based on a rapid and accurate diagnosis is therefore essential to prevent complications and eventually death (Kumar et al., 2009; Dellinger et al., 2013). Currently, positive blood cultures where bacteria are isolated from aseptically collected blood are considered as the gold standard to confirm sepsis (Fecteau et al., 1997a,b). However, their use is expensive and results are not immediately available (Fecteau et al., 1997a,b). To counteract this, a fast clinical sepsis score may be useful to help in the decision making of treatment in the field (Fecteau et al., 1997b). However, clinical signs often remain vague and difficult to differentiate from other neonatal diseases (Garcia et al., 2022), and sensitivity of a fast clinical sepsis score appears to be low (Pas et al., 2025). Therefore, a rapid, inexpensive, and reliable diagnostic test for sepsis would be valuable.

Calves suffering from sepsis present alterations in coagulation profile (Irmak et al., 2006). The most common abnormalities include changes in activated partial thromboplastin time, prothrombin time, fibrinogen, and fibrin and fibrinogen degradation products (Irmak et al., 2006). Viscoelastography, such as dynamic viscoelastic coagulometry, can be used in real time to measure the viscoelastic properties of the blood clot starting from the onset of formation to lysis (Hett et al., 1995) using point-of-care analyzers (Sonoclot, Sienco) and has several applications in human medicine, including sepsis detection (Schött and Björsell-Ostling, 1995). In veterinary medicine, the use of Sonoclot analysis has been described for horses (Jamieson et al., 2018), dogs (Babski et al., 2012), rabbits (Studer et al., 2021), chickens (Rodenbaugh et al., 2019), and owls (Leclerc et al., 2023), among others. Analysis time is also considered relatively rapid (within one hour) and inexpensive (approximately Can$5 [US$4] per sample).

Quantification of ATP activity by luminometry (in relative light units [**RLU**]) is a technique widely used in the medical and food industries to assess microbial contamination and hygiene as ATP is present in all living organisms, including bacteria (Corbitt et al., 2000). In cattle farming, ATP luminometry has been described for evaluating the cleanliness of colostrum-feeding equipment on the farm (Buczinski et al., 2022). Its main advantages are its rapid turnaround time, with results available within 15 s of sampling, and its low cost (under Can$5 per sample), making it potentially attractive for on-farm use. During sepsis, extracellular ATP concentrations are expected to increase markedly as a result of tissue damage, inflammation, and the presence of microorganisms (Ledderose et al., 2016), suggesting that ATP luminometry could serve as a rapid indirect indicator of sepsis. Similarly, dynamic viscoelastic coagulometry provides real-time information on coagulation dynamics and has shown possible diagnostic value in septic patients in human medicine (Schött and Björsell-Ostling, 1995), further supporting its potential relevance in neonatal calves.

The objective of this study was to establish reference blood values for ATP luminometry and dynamic viscoelastic coagulometry in healthy newborn calves. For ATP luminometry specifically, the study aimed to identify the sampling method and blood matrix (whole blood, serum, or plasma), with or without stabilizing solution, that would produce the lowest baseline RLU values in healthy individuals. Establishing such baseline conditions is essential to enable accurate discrimination between healthy calves and those with suspected sepsis in future studies. We hypothesized that normal reference values for both ATP luminometry and dynamic viscoelastic coagulometry would show low interindividual variability, that ATP RLU values would not differ between sampling methods, and that one blood matrix combined with a stabilizing solution would yield consistently low and stable baseline ATP measurements.

Sampling was performed from April 26 to 29, 2023, on healthy newborn (1–10 d old) dairy calves on farms that were clients of the ambulatory clinic at the Faculté de médecine vétérinaire de l'Université de Montréal. The study was approved by the Institutional Animal Care Committee (CÉUA) of the Université de Montréal (CÉUA protocol no. 22-Rech-2226). Because normal values for calves were unknown, no formal sample size calculation was possible. A total of 27 calves were sampled to account for any potential clinical or subclinical disease prevalence (∼25%) and ensure to include at least 20 healthy animals. This sample size was consistent with pilot veterinary studies and exploratory ATP luminometry research. Calves were examined to evaluate their health status, including respiratory rate, auscultation of the lungs, heart rate, rectal temperature, identification of possible diarrhea or umbilical infection, skin hydration status, capillary refill time, and suckling reflex. Because muscular movement may influence ATP luminometry levels (González-Alonso et al., 2002; Farias et al., 2005), blood sampling was performed before this clinical examination.

Figure 1 demonstrates an overview of the sampling procedure. In total, 22.5 mL of jugular blood was collected for each calf. For ATP luminometry measurements, both the sampling method and the collecting method were investigated to evaluate a practical approach that is applicable under real farm conditions. Regarding the sampling method, whole blood was drawn both aseptically (shaving and disinfecting the skin with chlorhexidine and 70% isopropyl alcohol and the use of sterile gloves) and by the conventional method. Sides were alternated per animal. Regarding blood matrices, whole blood, serum, and plasma were used. For whole blood and plasma, EDTA tubes were used to inhibit ATP catabolism (Gorman et al., 2007). Stabilizing solution was used only with whole blood and plasma because it stops ATP catabolism and inhibits cellular ATP release (Gorman et al., 2003) and would interfere with serum clot formation. Each tube was analyzed with 2 swabs according to the manufacturer's directions (Hygiena, Camarillo, CA): AquaSnap Total, which measures both microbial ATP (from living cells and particles), and AquaSnap Free, which measures free ATP (from nonmicrobial or dead cells). With this, microbial ATP can be calculated by subtracting free ATP from total ATP. Luminometry reading were immediately performed in duplicate to ensure repeatability. The time of blood collection and time of luminometry readings were noted for each blood matrix for each individual to indicate if time would have an influence on ATP measurements. The tube containing the stabilizing solution and plasma for each individual was kept for hemoglobin analysis in the laboratory. This was done because the amount of hemoglobin, which is an index of sample hemolysis, can increase the measured quantity of ATP (Gorman et al., 2007). By determining the quantity of hemoglobin, the ATP concentration can be corrected for the hemolysis component by using a formula (Gorman et al., 2007). Additionally, hematocrit concentration was determined in the blood of each individual to account for dilution.

**Figure 1:**
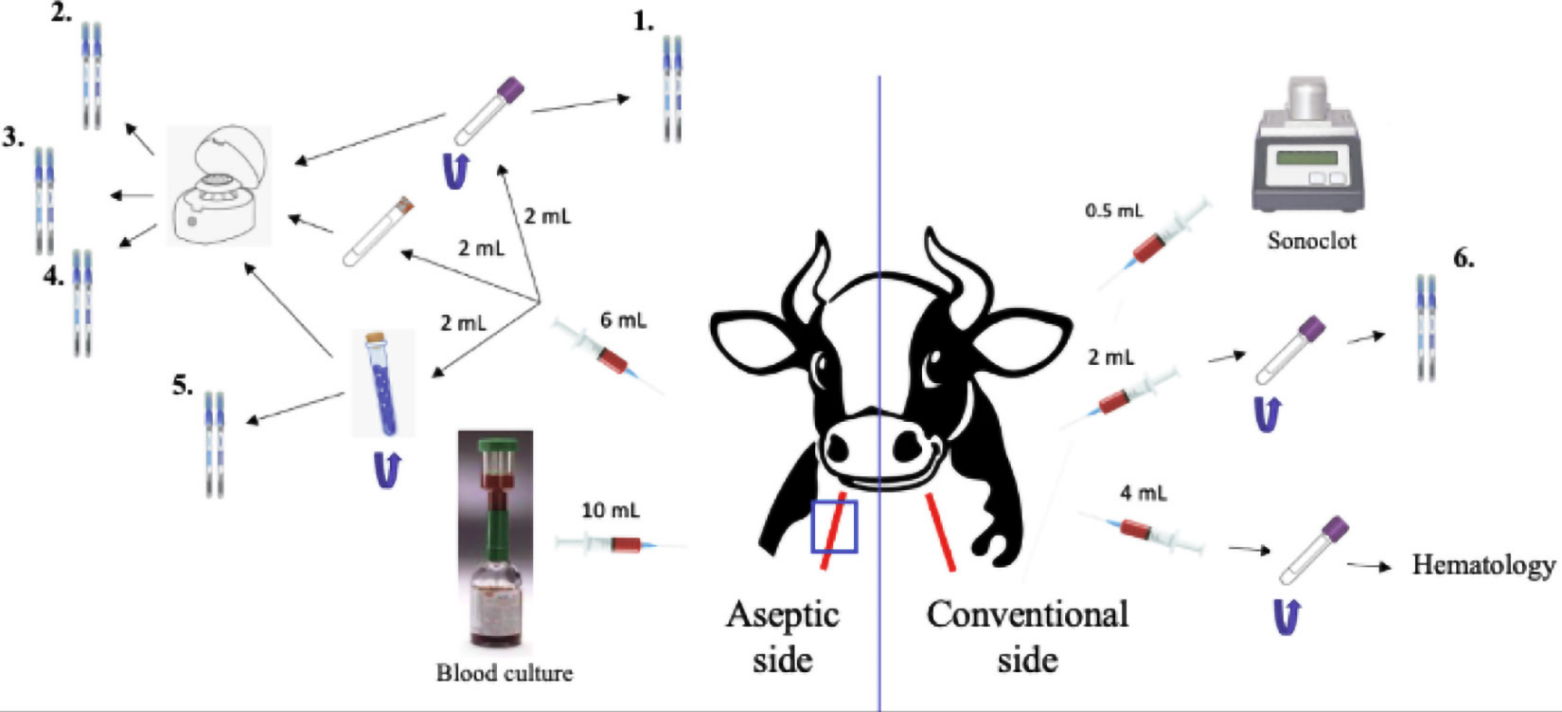
Overview of the sampling procedure. On the aseptic side, blood was collected with a syringe and inserted into tubes for luminometry examination (with or without centrifugation): (1) whole blood, (2) plasma, (3) serum, (4) plasma with stabilizing solution, and (5) whole blood with stabilizing solution. Two swabs were taken from each tube: AquaSnap Total and AquaSnap Free. The tube containing the stabilizing solution and plasma was kept for hemoglobin determination in the laboratory. Additionally, a blood culture sample was collected to verify sterility. On the conventional side, whole blood was collected for luminometry analysis (6; with AquaSnap Total and AquaSnap Free swabs), one sample for dynamic viscoelastic coagulometry analysis, and another for hematology blood analysis (hemoglobin, hematocrit, white blood cell count, and total proteins).

A blood culture (BACTEC Plus Aerobic medium, 8 to 10 mL; BD, Erembodegem, Belgium) was performed on the aseptic side as control for the detection of possible bacterial contamination (Marks et al., 2020). On the conventional side, whole blood was collected for analysis with the coagulation analyzer (Sonoclot, Sienco, Boulder, CO) using glass bed activator (0.5-mL tube, gbACT+ kit, Sienco, Boulder, CO). This analysis generates a clot formation curve and allows calculation of the activated clotting time (**ACT**), clot rate, and platelet function. The ACT indicates the time elapsed between sample activation and the appearance of fibrin. Clot rate represents the maximum slope of the Sonoclot signature during the initial clot formation, whereas platelet function quantifies the quality of clot retraction. Finally, a sample was collected and transferred into an EDTA tube to send to the laboratory for hemoglobin and hematocrit analysis, which can affect ATP results (Gorman et al., 2007), as well as for white blood cell count and total protein measurement to confirm that the animal was healthy. All luminometry and Sonoclot analyses were performed on the farm, and laboratory analyses were performed at the Diagnostic Veterinary Centre of the Université de Montréal (CDVUM, Saint-Hyacinthe, Québec, Canada).

Statistical analyses were performed using R software version 4.3.1 (R Development Core Team, 2020). Descriptive statistics were reported as the median and interquartile range (**IQR**). A Kruskal–Wallis nonparametric ANOVA and Dunn test with Benjamini-Hochberg correction for multiple pairwise comparisons were used to determine statistical differences in RLU values for the different sampling methods (aseptic whole blood or conventional whole blood) and blood matrices (serum, whole blood, plasma, whole blood with stabilizing solution, and plasma with stabilizing solution). The nonparametric Spearman correlation (Spearman's rho [**r_s_**]) was used to determine the correlation (with a fair correlation starting from r_s_ ≥ 0.30) between RLU values and hemoglobin and hematocrit values in whole blood, the correlation between RLU values and hemoglobin values in plasma with stabilizing solution, the correlation between RLU values and the time between blood collection and luminometry measurements, and the correlation between blood fibrinogen values and dynamic viscoelastic coagulometry parameters. A *P*-value <0.05 was set as the cut-off value for significance.

Six of the 27 calves sampled were excluded from the study due to an abnormal clinical examination (n = 4), a positive blood culture (n = 1), or both (n = 1), resulting in 21 calves included for this study (all female Holstein Friesian with a median age of 5 d, IQR of 3 to 8 d, with a normal white blood cell count, total protein measurement, and fibrinogen concentration). No significant difference was found between the aseptic method and conventional method for RLU values of whole blood (*P* = 0.47; Figure 2A). Furthermore, no significant difference was found in RLU values between the AquaSnap Total and AquaSnap Free samples for the aseptic method (*P* = 0.7) and the AquaSnap Total and AquaSnap Free samples for the conventional method (*P* = 0.5). A comparison of the RLU values between the different blood matrices (with and without stabilizing solution) can be found in Figure 2B. Within each blood matrix, no significant difference could be found between the AquaSnap Total and AquaSnap Free samples (*P* > 0.3). For measurements obtained with the AquaSnap Total swab, significant differences were observed among blood matrices (*P* < 0.001; Figure 2B). Specifically, pairwise comparisons revealed significant differences between (1) plasma and whole blood, (2) serum and whole blood, (3) plasma and whole blood with the stabilizing solution, (4) serum and whole blood with the stabilizing solution, (5) serum and plasma with the stabilizing solution, and (6) whole blood with the stabilizing solution and plasma with the stabilizing solution. AquaSnap Free swab RLU results showed significant overall differences between blood matrices (*P* < 0.001; Figure 2B), with significant pairwise differences identified between (1) serum and whole blood, (2) plasma and whole blood with stabilizing solution, (3) serum and whole blood with stabilizing solution, and (4) whole blood with stabilizing solution and plasma with stabilizing solution.

**Figure 2:**
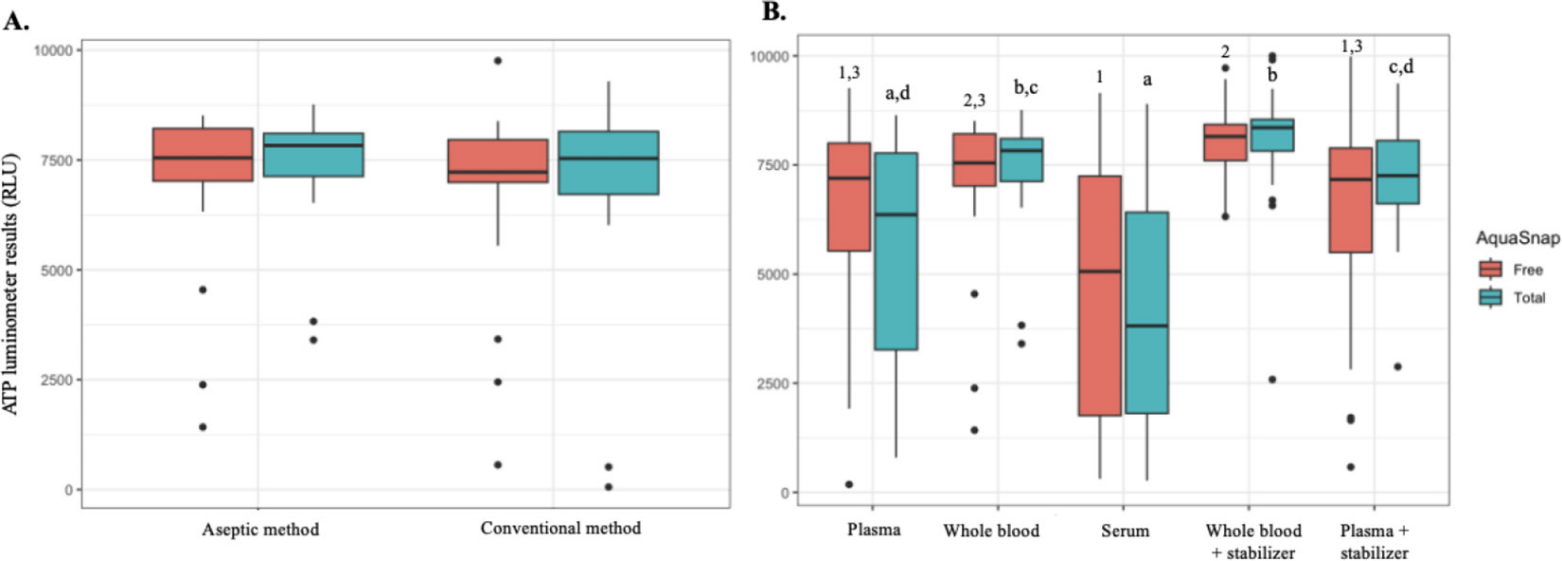
Adenosine triphosphate luminometer results (expressed in relative light units [RLU]) comparing the sampling method (A) and blood matrices (B) from 21 newborn dairy calves, demonstrated as boxplots representing the median (midline) and interquartile range (upper and lower box edges). Whiskers are defined as 1.5 × IQR, and dots are defined as outliers. Both AquaSnap Free and AquaSnap Total results are displayed. No significant difference in RLU values was seen between the aseptic and conventional sampling method (A; *P* = 0.47). For the sampling medium (B), a significant difference (*P* < 0.001) between blood matrices is noted by different numbers (1–3) for the AquaSnap Free results, and different letters (a–d) for the AquaSnap Total results.

A negative correlation was observed between hemoglobin and RLU values in whole blood (r_s_ = −0.40; *P* = 0.008). The correlation between hemoglobin and RLU values in whole blood with stabilizing solution was also examined, yielding a negligible and nonsignificant relationship (r_s_ = 0.05; *P* = 0.83). A negative correlation was noted between hematocrit and RLU values in whole blood (r_s_ = −0.44; *P* = 0.004), whereas no correlation was found between hematocrit and RLU values in whole blood with stabilizing solution (r_s_ = 0.013; *P* = 0.95). Finally, the potential influence of time (time between blood collection and luminometry analysis) on RLU values was assessed, with a median time difference of 25 min (minimum of 6 min and maximum of 69 min, as coagulation time was required for serum analysis). This correlation was negligible and not significant (r_s_ = −0.06; *P* = 0.7).

Regarding the dynamic viscoelastic coagulometry analysis, 19 normal blood values could be obtained (for 2 healthy individuals, no results were available due to analyzer error). The median (interquartile range) for ACT was 172 s (154–192), 25 units/min (18–34) for clot rate, and 3.30 units (2.70–3.90) for platelet function (n = 19). In addition, no correlation was detected between blood fibrinogen and dynamic viscoelastic coagulometry parameters (ACT: r_s_ = 0.21, *P* = 0.41; clot rate: r_s_ = 0.03, *P* = 0.92; and platelet function: r_s_ = 0.10, *P* = 0.70).

The objective of this study was to determine reference blood values of healthy newborn calves for both ATP luminometry and dynamic viscoelastic coagulometry analysis. This information would help determine if one or both techniques would have the potential in later studies to indicate sepsis in calves in the field. Regarding luminometry results, no significant difference in RLU values between the AquaSnap Total and AquaSnap Free samples was found in our study. Because no bacterial contamination was present in the blood samples, it should be expected that these values would be in a similar range. It is surprising that values of the AquaSnap Free samples exceeded the values of the AquaSnap Total samples as we observed in this study for serum and plasma samples, although this difference was not significant and was probably due to interference or artifacts. Sepsis can reduce intracellular ATP due to mitochondrial dysfunction (Preau et al., 2021) and increase extracellular ATP due to tissue damage and inflammation (Ledderose et al., 2016). Thus sepsis would, in theory, increase the AquaSnap Free test results, but because AquaSnap Total equals intracellular ATP plus free ATP, the AquaSnap Total test results would not decrease. We found no significant difference in RLU values between the conventional method and aseptic method of sampling. These results would favor using the conventional method because it is more practical, faster, and cheaper in the field.

Different blood matrices were investigated to determine the method that would yield the lowest and least variable RLU baseline values. Significantly different RLU values between the different blood matrices were observed in our study, both for the AquaSnap Total and the AquaSnap Free samples. Overall, serum and plasma samples provided the lowest RLU values in this study, probably due to the lack of a cellular component as an ATP source. Time between sampling and sample analysis was not shown to be a contributing factor to these values in the current study. However, values remained relatively high considering the upper detection limit of the luminometer machine (i.e., 10,000 RLU) and exhibited considerable variability, preventing the establishment of narrow reference intervals suitable for potential diagnostic application under septic conditions in calves. In literature, ATP plasma values of 1,000 nmol/L and 31 nmol/L have been described in humans and dogs, respectively (Farias et al., 2005; Gorman et al., 2007). It must be noted that these studies mention ATP values obtained by the Berthold (Oak Ridge, TN) LB 9507 luminometer, which differs from the luminometer used in the current study (Hygiena, Camarillo, CA). Measuring ATP levels in nanomoles per liter expresses the actual amount of ATP molecules present in a sample and thus gives an absolute quantitative value, whereas RLU is a raw luminescence signal that measures the light intensity emitted in a bioluminescent ATP assay and thus gives a relative, instrument-dependent value. Both values have a very strong correlation (r = 0.99), with RLU values being consistently significantly higher (in the order of ×10^5^) than ATP values (Gorman et al., 2007; Younès et al., 2011). Future studies could consider other luminometer machines which perhaps could give better outcomes, although the price and practicality in the field need to be taken into account. In an attempt to lower ATP concentrations, stabilizing solution was used in our study, which has been shown to significantly decrease ATP values (Gorman et al., 2007). No significant difference in RLU values could be found between samples with or without stabilizing solution in this study, both for plasma as for whole blood, indicating that stabilizing solution does not contribute to a reduction in RLU values in blood of healthy calves. Possible explanations are that RLU values are of a too high an order to be able to reduce comparison with ATP values, that the dose used was not sufficient for this species and technique, or perhaps due to a different metabolism seen in calves.

Because an elevated hemoglobin concentration and hematocrit percentage (hemoconcentration) may increase measured ATP levels (Gorman et al., 2007), both were determined and a correlation with RLU values was calculated. A moderate negative correlation was found between hemoglobin and hematocrit concentrations and RLU values in whole blood, whereas no correlation was found between those 2 factors and RLU values in stabilized solution in our study. This is in contrast with what has been described in the literature (Gorman et al., 2007) and could be due to a different methodology (whole blood in our study instead of an erythrocyte pellet), different measuring unit (RLU in our study instead of ATP), or physiological differences in blood between calves and humans (including the fact that prey animals are likely to be more stressed when being hold for taking blood compared with humans), or because higher hematocrit leads to a darker solution that absorbs some of the photons and prevents them from reaching the detector.

Regarding the dynamic viscoelastic coagulometry reference values established in healthy newborn calves, the observed low interindividual variation is favorable for developing reference intervals using this method. However, only 19 individuals were included in this study. Reference intervals are generally established only when more than 20 samples are available, and ideally 120 samples (Friedrichs et al., 2012). Therefore, additional sampling would be required to have a more robust idea and higher statistical power of the true reference values in the blood of healthy newborn dairy calves. No correlation between fibrinogen and dynamic viscoelastic coagulometry parameters could be found in our study, which contrasts with canine and human studies, particularly for clot rate– and clot strength–related variables (Babski et al., 2012; Espinosa et al., 2014). The latter 2 studies used the Clauss assay on citrated plasma, the functional gold standard for fibrinogen quantification, whereas our study used EDTA-based measurements, which do not reflect functional fibrinogen activity. This methodological difference represents a limitation of our work, and future studies should use Clauss fibrinogen to allow appropriate comparison with Sonoclot data. Although the turnaround time with dynamic viscoelastic coagulometry analysis is longer compared with ATP luminometry (a couple of minutes compared with 15 s), it is still considerably more rapid compared with the current method of blood culture that takes several days. Additionally, further research including calves in sepsis would be necessary to determine if dynamic viscoelastic coagulometry analysis would be suitable as a fast, economical on-farm technique to determine possible sepsis in calves.

To conclude, ATP luminometry currently demonstrated methodological constraints that would discourage further research. The reason for this is that RLU values are generally too high or show excessive variability among healthy animals, with no decrease of values with stabilizing solution and no usable correction with hemoglobin and hematocrit concentration in the blood. Conversely, dynamic viscoelastic coagulometry values showed low interindividual variability in healthy calves, encouraging further research with septic calves to determine its potential as an indicator for rapid sepsis detection.
